# Facial expression adaptation impairs perceived social signal across expressions

**DOI:** 10.1007/s00426-025-02094-4

**Published:** 2025-03-24

**Authors:** Kazusa Minemoto, Yoshiyuki Ueda, Sakiko Yoshikawa

**Affiliations:** 1 Institute for the Future of Human Society, Kyoto University, Kyoto, Japan; 2 Faculty of the Arts, Kyoto University of the Arts, Kyoto, Japan

**Keywords:** Facial expression, Adaptation, Social signal, Motivation, Mood, Perception

## Abstract

Facial expressions provide crucial cues for assessing others’ internal states, leading to appropriate responses to maintain social harmony. This study investigated how social signals evoked by facial expressions are perceived, focusing on their automatic triggering and relationship to the representations of the expressions by using adaptation aftereffect paradigm. Participants evaluated their perceived need for help and their motivation to provide help in response to sad and fearful expressions, both of which convey help signals, before and after adaptation. Accordingly, the ratings for perceived need for help for both sad and fearful expressions decreased following adaptation to sad expressions, indicating that perceived need for help was represented independently from the perception of test expressions. Additionally, this result also indicates that the need for help is automatically triggered during viewing sad facial expressions. However, the ratings for perceived need for help for fearful expressions alone decreased after adaptation to fearful expressions. The motivation to help remained unaffected by adaptation to sad or fearful expressions. Additional experiments demonstrated that impairment of perceived need for help occurs independently of the perceived intensity of the expressions and participants’ moods. This study suggests that social signals can be represented independently from facial expressions, and facial expression adaptation paradigms can examine this possibility.

**Supplementary Information:**

The online version contains supplementary material available at 10.1007/s00426-025-02094-4.

## Introduction

In everyday interactions, accurately understanding others’ internal states is crucial for fostering social harmony. Facial expressions serve as significant nonverbal cues for efficient estimation of these states, prompting appropriate responses (Van Kleef & Côté, 2022). This study investigates the perception of social signals evoked by facial expressions, specifically examining whether observing facial expressions simultaneously triggers perception of social signals (i.e., automaticity of that process) and how they relate to the representations of the expressions.

We employed the adaptation aftereffect paradigm to conduct this examination. Adaptation, an automatic function that optimizes perception, enhances sensitivity to changing stimuli while reducing it to unchanging ones (Pestilli et al., 2007). This function applies not only to simple features like color, line orientation, and movement but also to complex objects such as faces (identity, ethnicity, gender, attractiveness, and expressions) and body language (Fedorov et al., 2018; Hsu & Young, 2004; Leopold et al., 2001; Minemoto & Ueda, 2022; Webster et al., 2004; Winkler & Rhodes, 2005). Adaptation has also been instrumental in examining neural representations of face recognition. For instance, Fox and Barton (2007) discovered that a neural representation of facial expression is independent from the identity of facial expression expressers by showing that facial expression adaptation aftereffects occurred even when the identities of photo models differed between the adaptation and test stimuli.

A similar relationship might exist between facial expressions and their social signals. That is, there may be a representation concerning social signals common among multiple facial expressions (facial expression-independent). This can be investigated using different facial expression categories conveying the same social signals between adaptation stimuli and test stimuli. Typically, aftereffect on the perception of facial expressions is selectively observed within the same expression category. For example, stronger intensities of happy expressions are required to perceive happiness after adapting to happiness (Hsu & Young, 2004; Webster et al., 2004). However, no or opposite effects are observed across different categories of expressions between adaptation and test stimuli, such as happy and sad (Hsu & Young, 2004; Juricevic & Webster, 2012). If the sensitivity to social signals evoked by facial expressions is common among multiple facial expressions (i.e., facial expression-independent), the perception of these signals could be impaired by adaptation, even if they are evoked by different facial expressions.

Previous research has explored the signals of trustworthiness and friendliness derived from facial expressions (Engell et al., 2010; Prete et al., 2018). Engell et al. (2010) investigated the shared neural systems of trustworthiness and facial expressions, utilizing two expressions with contrasting effects on trustworthiness (happiness and anger) and one with no effect (fear). The study found that trustworthiness evaluations of neutral expressions increased after adapting to angry expressions, decreased after adapting to happy expressions, and remained unaffected after adapting to fearful expressions. This suggests that overlapping neural populations of trustworthiness and expressions may mutually influence each other. Prete et al. (2018) used angry and happy expressions and found that their perception influences friendliness, and that adaptation to these expressions also affects evaluations of trustworthiness and friendliness. However, it is premature to assert that the relationship between the perception of facial expressions and perceived social signals has been thoroughly examined. This is because happy expressions are associated with higher levels of trustworthiness and friendliness, while angry expressions are associated with lower levels of both. This implies a simple correspondence: as the intensity of happy expressions or positive impressions decreases and the intensity of angry expressions or negative impressions increases, perceived trustworthiness and friendliness decrease.

To address these issues, this study employed sad and fearful expressions, which are associated with signals of help. Helping behavior, which establishes cooperative relationships within a group and facilitates adjustment to changing environments, is observed at an early stage of human development (i.e. infancy; Warneken & Tomasello, 2006), indicating its fundamental importance for human survival. Sad and fearful expressions are universally recognized (Ekman et al., 1969), and their recognition is linked to prosocial behavior or altruism (Marsh et al., 2007, 2014; Marsh & Ambady, 2007; Nakashima et al., 2009). Compared to neutral expressions, these two expressions are highly rated in terms of prosocial responding. Nakashima et al. (2009) examined the social signals concerning helping behaviors using sad and fearful facial expressions and reported that the perception of the need for help was comparable for both expressions. On the other hand, when it comes to motivation to provide help, which is a complicated decision-making process as providing help demands commitment, rather than a simple perception of the need for help, there was a difference between the two facial expressions; the fearful facial expression received a lower motivation to help. Considering that these evaluation values for both expressions for sadness and fear were higher than for neutral expressions, it is suggested that although sad and fearful facial expressions are related to social signals concerning helping behaviors, they are not exactly the same.

The aim of this study was to investigate whether exposure to facial expressions alters the perception of these social signals. We used two different facial expressions, sadness and fear, which are both associated with the same signal: need for help. In addition, we also examined the motivation to provide help to investigate the extent to which adaptation influences our judgment. The first hypothesis posited that the perceived social signal is directly determined by the perceived facial expressions (a facial expression-dependent manner). Since previous findings that adapting to facial expressions impair the perceived intensity of the same facial expression (Hsu & Young, 2004; Juricevic & Webster, 2012), the perception of the facial expression-dependent social signals would be suppressed only after the adaptation of the same facial expressions. Therefore, the adaptation of helping judgment should only be observed when the categories of facial expressions in the testing stimuli match those in the adaptation stimuli. The second hypothesis proposed the existence of a general helping signal representation across facial expressions. While the category of facial expression is an important cue for social signals (Marsh & Ambady, 2007; Nakashima et al., 2009), there may be both facial expression-dependent and facial expression-independent representations of the helping signal. For instance, a study exploring the representation of identity and facial expressions using adaptation suggested the existence of identity-dependent and identity-independent representations of expression (Fox & Barton, 2007). If the facial expression-independent representation of helping is valid, exposure to sad facial expressions will suppress perceived helping signals not only for people with sad facial expressions but also for those with fearful expressions. Similarly, exposure to fearful facial expressions will suppress helping signals for people with both sad and fearful facial expressions.

In Experiment 1, participants adapted to sad facial expressions and evaluated their perception of the need for help and motivation to provide help using a seven-point Likert scale before and after adaptation. Lower ratings after exposure, compared to before exposure, can be interpreted as the occurrence of an aftereffect. In Experiment 2, participants adapted to fearful facial expressions instead of sad facial expressions.

### Experiment 1

This experiment investigated if the perceived necessity to help, and the motivation to help persons displaying sad, fearful, and neutral expressions, diminished following adaptation to sad facial expressions.

## Method

### Participants

Eighteen undergraduate and graduate students (six of whom were women; average age 21.8 years, SD = 4.0), with normal or corrected-to-normal vision, participated in the experiment. All participants were naïve to the experiment’s purpose. Previous studies using the adaptation paradigm typically had 10 or fewer participants for each condition (Fox & Barton, 2007; Leopold et al., 2001; Webster et al., 2004). These studies employed discrimination tasks, whereas the current study used rating tasks, which might potentially exhibit larger individual differences. Consequently, approximately twice as many participants as in previous studies were included (i.e., 18). Once 18 participants had been recruited, no further additions or collections were made. All participants provided signed consent prior to the experiment, which adhered to the principles of the Declaration of Helsinki.

### Apparatus and stimuli

Each participant completed the experimental trials individually. The stimuli were displayed on a 19-inch DELL Trinitron P992 monitor (refresh rate 85 Hz, spatial resolution 1024 × 768 pixels) using the Windows 7 operating system and SuperLab 2.0 software (Cedrus). Participants’ heads were positioned 40 cm from the monitor. Five Japanese female models exhibiting neutral, sad, or fearful facial expressions were selected from the Advanced Telecommunications Research Institute International, Japan, database set. For each model, two intensities (strong and weak) of sad and fearful facial expressions were created by morphing each facial expression with the neutral expression. The use of two intensities aimed to diversify the rating scores of the test stimuli, preventing participants from directly associating rating scores with facial expression categories. To standardize the intensity of the facial expressions across models, twelve participants in a pilot study judged the intensity of the morphed expressions using a Likert scale (from 1: very weak to 7: very strong). This process determined the strong and weak facial expressions used in the experiments. The mean ratings of the strong and weak sad expressions used in the experiments were 5.5 (SD = 0.58) and 3.7 (SD = 0.46), respectively, while those of the strong and weak fearful expressions were 5.7 (SD = 0.35) and 3.6 (SD = 0.27), respectively. All photo stimuli measured 11.9° × 9.7° in visual angle.

One of the original sad facial expressions of the five models (i.e., original picture) was used as the adaptation stimulus. The test stimuli consisted of 25 images, which included the five models’ strong and weak facial expressions of sadness and fear, as well as neutral facial expressions. Thus, each participant was exposed to one adaptation stimulus and 25 test stimuli.

### Procedure

**Fig. 1:**
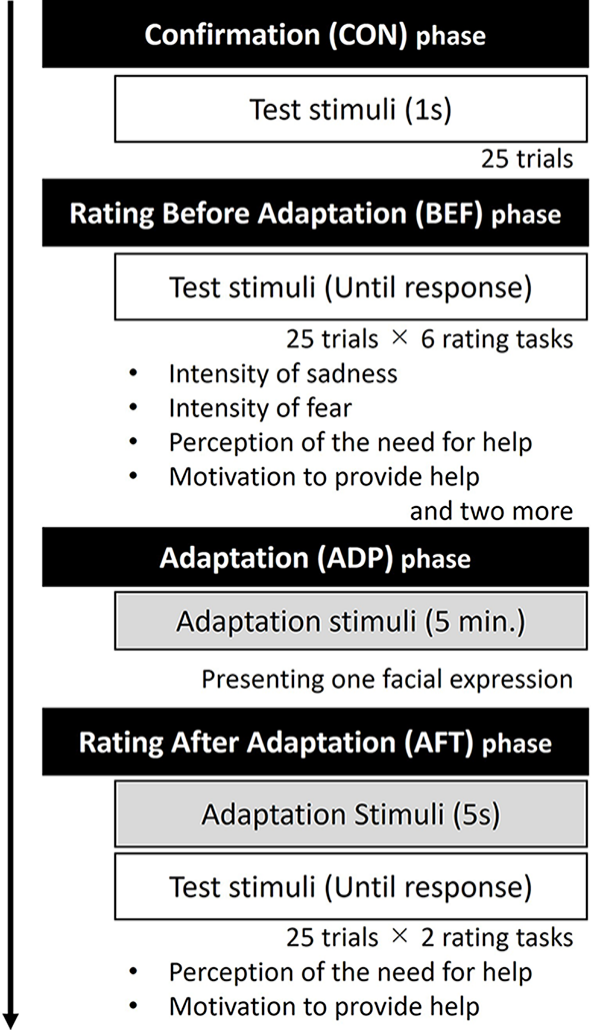
Flowchart of the Experiment 1: It consists of Confirmation (CON) phase, Rating before adaptation (BEF) phase, Adaptation (ADP) phase, and Rating after adaptation (AFT) phase

The experiment consisted of four phases: confirmation, rating before adaptation rating, adaptation, and rating after adaptation rating. All test stimuli were presented once in each phase, excluding the adaptation phase, resulting in 25 trials within a single block. The flowchart of the experiment is shown in Fig. 1.

*Confirmation (CON) phase*. All stimuli were sequentially displayed one-by-one at the screen’s center in random order. Each stimulus was presented for 1 s. Participants were instructed to observe them in preparation for rating in subsequent phases.

*Rating before adaptation (BEF) phase*. Participants were asked to envision a scenario where they were interacting with the individual in the photograph and rate the images based on the following: “How much do you think this person needs your help?” (perception of the need for help), “How much do you want to help this person?’ (motivation to provide help), “How much sadness does this person feel?” (intensity of sadness), “How much fear does this person feel?” (intensity of fear), and two more items on a seven-point Likert scale.^1^ These instructions were provided at the start of each block, followed by the presentation of all test stimuli in a random order at the screen’s center. Participants responded using the corresponding number keys on a keyboard. The stimuli were displayed until a key was pressed, and the subsequent trial commenced 1 second after the press.

*Adaptation (ADP) phase*. The adaptation stimulus was displayed at the screen’s center for 5 min. Participants were instructed to maintain their gaze on the stimulus and avoid intentionally closing their eyes.

*Rating after adaptation (AFT) phase*. Test stimuli were individually presented in a random order, and participants rated their perception of the need for help and their motivation to provide help again. Therefore, only two blocks (i.e., perception of the need for help and motivation to provide help) were conducted in this phase. The instructions and rating scales were identical to those in the BEF phase. To maintain the adaptation aftereffect, the adaptation stimulus was displayed for 5 s before each test stimulus presentation (Rhodes et al., 2010). In this phase, the inter-stimulus interval was 100 ms between the adaptation stimulus and the subsequent test stimulus, and the test stimuli were displayed for 200 ms. The next trial began 1 s after the participant’s response. Participants were explicitly instructed to rate the presented test stimuli independently, without comparing them to the adaptation stimulus or striving to maintain consistency with the BEF phase ratings.

### Analysis

We conducted analyses for three major dependent variables: intensity of sadness and fear, perception of the need for help, and motivation to provide help. To calculate the dependent variables for all analyses, we averaged the rating scores for each facial expression (i.e., neutral, sad, and fearful) for all rating items in each phase (“intensity of sadness” and “intensity of fear” in the BEF phase, and “perception of the need for help” and “motivation to help” in BEF and AFT phases). As there was no specific hypothesis regarding the intensities of test facial expressions, they were collapsed in the analyses. Initially, to verify whether participants accurately recognized the facial expressions of the test stimuli, the average ratings of the recognized intensity of sadness and fear for each facial expression in the BEF phase were analyzed using a one-way repeated measures analysis of variance (ANOVA) with three levels of facial expressions (neutral, sad, and fearful). In this analysis, dependent variables are the mean ratings of intensity of sadness or fear in BEF and independent variable was facial expressions (neutral, sad, and fearful). Subsequently, to examine the aftereffect, a two-way repeated measures ANOVA with phase (BEF vs. AFT) and facial expression (sad vs. fearful vs. neutral) was conducted for the average ratings of the need for help and motivation to provide help. In this analysis, dependent variables are the mean ratings of the need for help and motivation to provide help and independent variables were phase (BEF and AFT) and facial expressions (neutral, sad, and fearful).

### Data collection and availability

The raw data for this experiment can be found at https://osf.io/3zrfb/. The materials are available upon request, as they contain personal information (faces).

## Results

### Intensity of sadness and fear

**Fig. 2:**
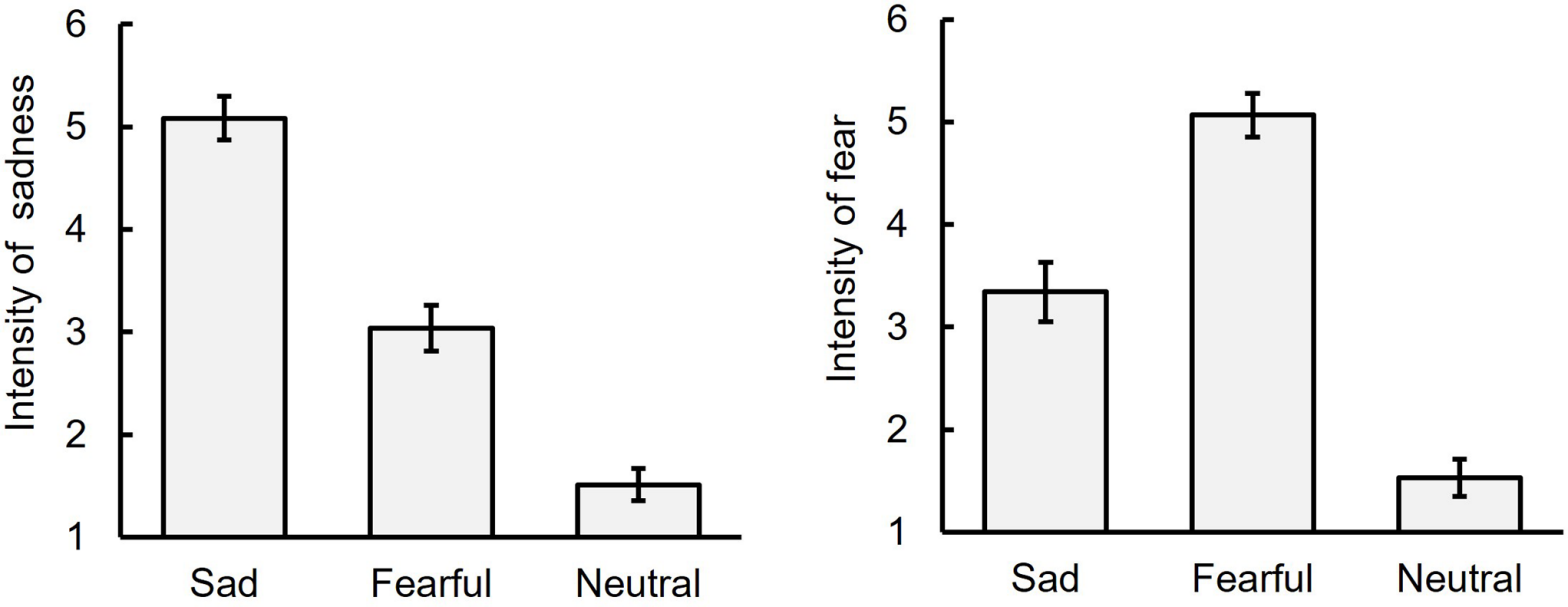
Intensity ratings of sadness and fear for sad, fearful, and neutral facial expressions before the adaptation phase (Experiment 1). Note: The error bar indicates standard error

Figure 2 presents the mean ratings of intensities for sadness and fear across three facial expressions (neutral, sad, and fearful) during the BEF phase (see Table S1 in Online Resource for descriptive statistics). A one-way repeated measures ANOVA revealed a significant main effect of facial expression on the intensity of sadness, *F* (2, 34) = 114.31, *p* <.001, η_p_^2^ = 0.87. Ryan’s method for multiple comparisons indicated that sad facial expressions received the highest ratings, followed by fearful and neutral expressions, *t*s (34) ≥ 6.44, *p* <.001, *r*s *≥* 0.74. Similarly, for the intensity of fear, a significant main effect of facial expression was observed, *F* (2, 34) = 80.92, *p* <.001, η_p_^2^ = 0.83. Fearful facial expressions were rated highest, followed by sad and neutral expressions, *t*s (34) ≥ 6.20, *p* <.001, *r*s *≥* 0.73. These findings confirm the correct recognition of facial expressions by the participants.

### Perception of the need for help

**Fig. 3:**
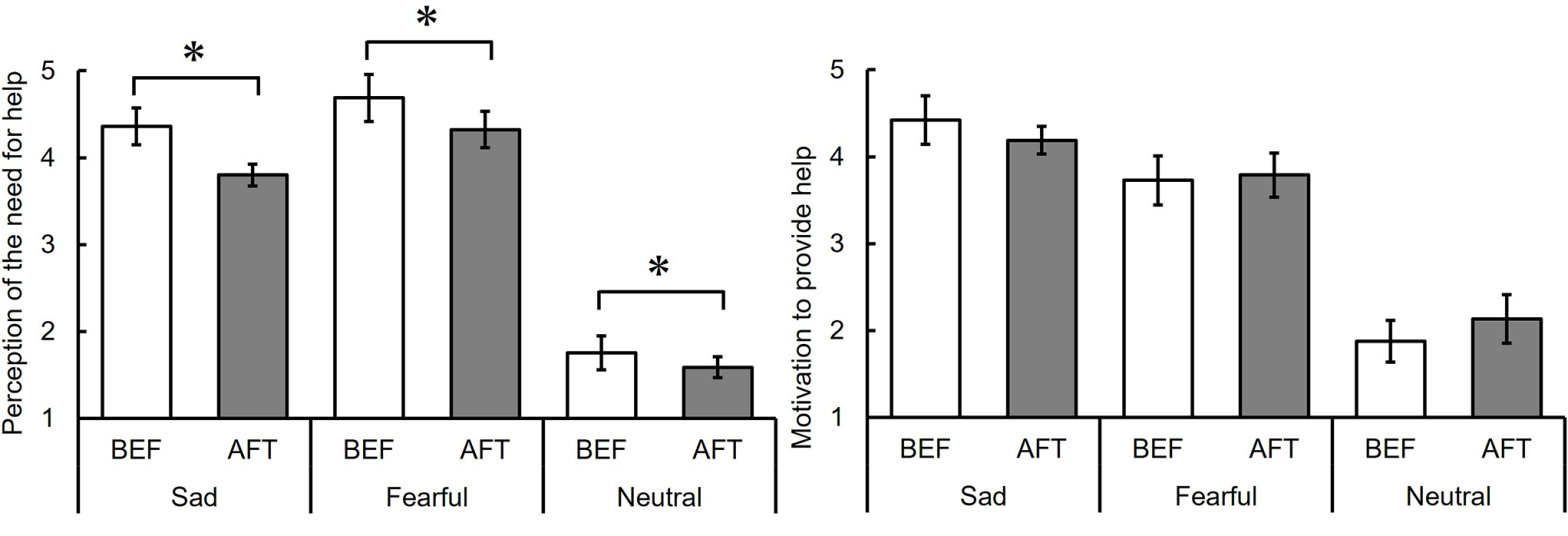
Ratings of the perception of the need for help and motivation to help before (BEF) and after (AFT) adaptation to sad facial expressions for sad, fearful, and neutral facial expressions (Experiment 1). Note: The error bar indicates standard error. The asterisk indicates significant differences between BEF and AFT

Figure 3 displays the mean ratings of perceived need for help for individuals exhibiting sad, fearful, and neutral facial expressions (see also Table S2). A two-way repeated measures ANOVA with phase (BEF vs. AFT) and facial expression (sad vs. fearful vs. neutral) yielded a significant main effect of phase, *F* (1, 17) = 5.66, *p* =.03, η_p_^2^ = 0.25^2^, suggesting a decrease in the perceived need for help in the AFT phase compared to the BEF phase. Additionally, a significant main effect of facial expression was found, *F* (2, 34) = 96.77, *p* <.001, η_p_^2^ = 0.85. Subsequent multiple comparisons showed that the ratings for sad and fearful facial expressions exceeded those for the neutral expression, *t* (34) = 10.96, *p* <.001, *r* =.88 and *t* (34) = 12.90, *p* <.001, *r* =.91, respectively, with comparable ratings for sad and fearful expressions, *t* (34) = 1.94, *p* =.06, *r* =.34. No significant interaction between facial expression and phase was detected, *F* (2, 34) = 1.95, *p =*.16, η_p_^2^ = 0.10. These results suggest that adaptation to sad facial expressions diminished the perception of the need for help for individuals with both sad and fearful expressions.

### Motivation to provide help

Figure 3 also illustrates the mean ratings of participants’ motivation to help individuals with sad, fearful, and neutral facial expressions (see also Table S2). A two-way repeated measures ANOVA with phase (BEF vs. AFT) and facial expression (sad vs. fearful vs. neutral) revealed a significant main effect of facial expressions, *F* (2, 34) = 33.13, *p* <.001, η_p_^2^ = 0.66. Further multiple comparisons showed that the ratings for sad and fearful facial expressions surpassed those for the neutral expression, *t* (34) = 7.79, *p* <.001, *r* =.83, *t* (34) = 5.94, *p* <.001, *r* =.75, respectively, with similar ratings for sad and fearful expressions, *t* (34) = 1.85, *p* =.07, *r* =.34. Neither a main effect of phase nor an interaction between facial expression and phase was significant, *F* (1, 17) = 0.02, *p* =.88, η_p_^2^ = 0.00, *F* (2, 34) = 1.56, *p =*.23, η_p_^2^ = 0.07, respectively. These findings suggest that adaptation to sad facial expressions did not influence the motivation to help.

## Discussion

In Experiment 1, we investigated the impact of adaptation to sad facial expressions on judgments related to helping, specifically the perception of the need for help and the motivation to provide help. The BEF phase revealed that participants accurately recognized the presented facial expressions, as indicated by the intensity of perceived sadness and fear. Furthermore, participants rated the need for help and the motivation to help higher for individuals displaying sad and fearful facial expressions compared to those with neutral expressions, demonstrating an understanding of the evaluation criteria.

Interestingly, the perception of the need for help declined after adaptation to sad facial expressions, irrespective of whether the test stimulus displayed a sad or fearful expression. This finding contradicts previous studies on facial expression recognition adaptation, which reported a selective suppression pattern of adaptation aftereffects within the same facial expression category across the adaptation and test stimuli (Hsu & Young, 2004; Juricevic & Webster, 2012). These results suggest that the decrease in the perceived need for help may not be attributed to a reduced sensitivity to facial expressions. Instead, it appears that adaptation to sad facial expressions suppresses the perceptual mechanism associated with the need for help. Furthermore, the perception of the need for help was also impaired in neutral facial expressions, suggesting that adaptation to sad facial expressions suppressed the perception of the need for help not only for facial expressions related to helping (i.e., sad and fearful facial expressions) but also for other facial expressions.

The motivation to help remained unchanged following adaptation to sad facial expressions, indicating that the intensity of facial expressions was not the primary determinant of participants’ motivation to help. Instead, this motivation may be influenced by the category of facial expressions, as the ratings for sad and fearful facial expressions were significantly higher than those for neutral expressions.

### Experiment 2

Experiment 1 demonstrated that adapting to sad facial expressions inhibited the perceptual recognition of help necessity. To determine if this effect extends to all expressions associated with helping, Experiment 2 investigated whether the perception of help necessity and the motivation to help individuals displaying sad, fearful, and neutral facial expressions diminished following adaptation to fearful expressions.

## Method

### Participants

Eighteen undergraduate and graduate students, seven of whom were women (mean age 20.5; SD = 2.0), with normal or corrected-to-normal vision, participated. All participants were naïve to the experiment’s purpose. All participants provided signed consent prior to the experiment, which adhered to the principles of the Declaration of Helsinki.

### Apparatus, stimuli, and analysis

The apparatus, stimuli, and analysis were identical to those in Experiment 1.

### Procedure

The procedure mirrored that of Experiment 1, with the exception that fearful facial expressions were utilized as adaptation stimuli in place of sad facial expressions.

### Data collection and availability

The raw data for this experiment can be found at https://osf.io/3zrfb/. Materials are available upon request to the corresponding author because, as they contain personal information (faces).

## Results

### Intensity of sadness and fear

**Fig. 4:**
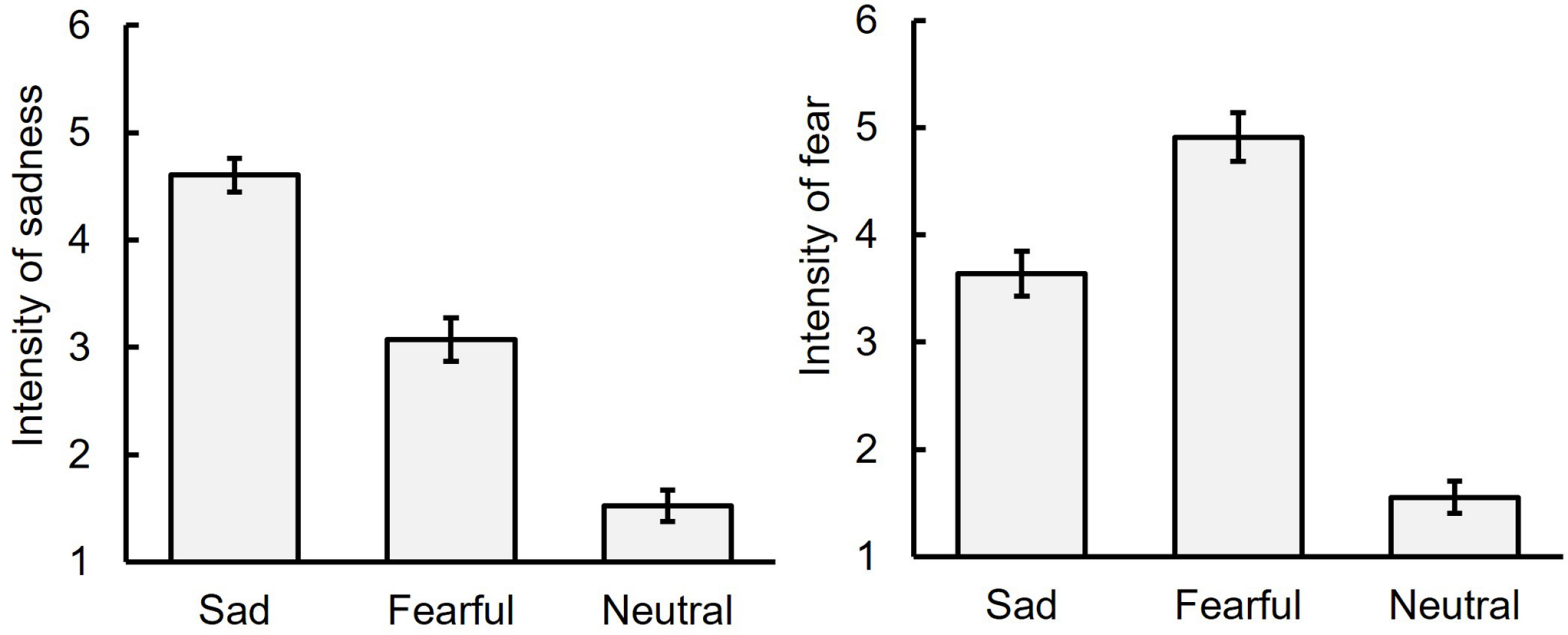
Intensity ratings of sadness and fear for sad, fearful, and neutral facial expressions before the adaptation phase (Experiment 2). Note: The error bar indicates standard error

Figure 4 presents the average ratings of sadness and fear intensity for each facial expression (neutral, sad, and fearful) in the BEF phase (see also Table S3). A one-way repeated measures ANOVA revealed a significant main effect of facial expression on the intensity of sadness, *F* (2, 34) = 129.91, *p* <.001, η_p_^2^ = 0.88. Multiple comparisons indicated that the rating for sad facial expressions was the highest, followed by fearful and neutral expressions, *t*s (34) ≥ 8.02, *p* <.001, *r*s *≥* 0.81. For the intensity of fear, a significant main effect of facial expression was also observed, *F* (2, 34) = 189.49, *p <*.001, η_p_^2^ = 0.92. Multiple comparisons revealed that the rating for fearful facial expressions was the highest, followed by sad and neutral expressions, *t*s (34) ≥ 7.31, *p* <.001, *r*s ≥ 0.78. These results suggest that the participants correctly recognized the models’ facial expressions.

### Perception of the need for help

**Fig. 5:**
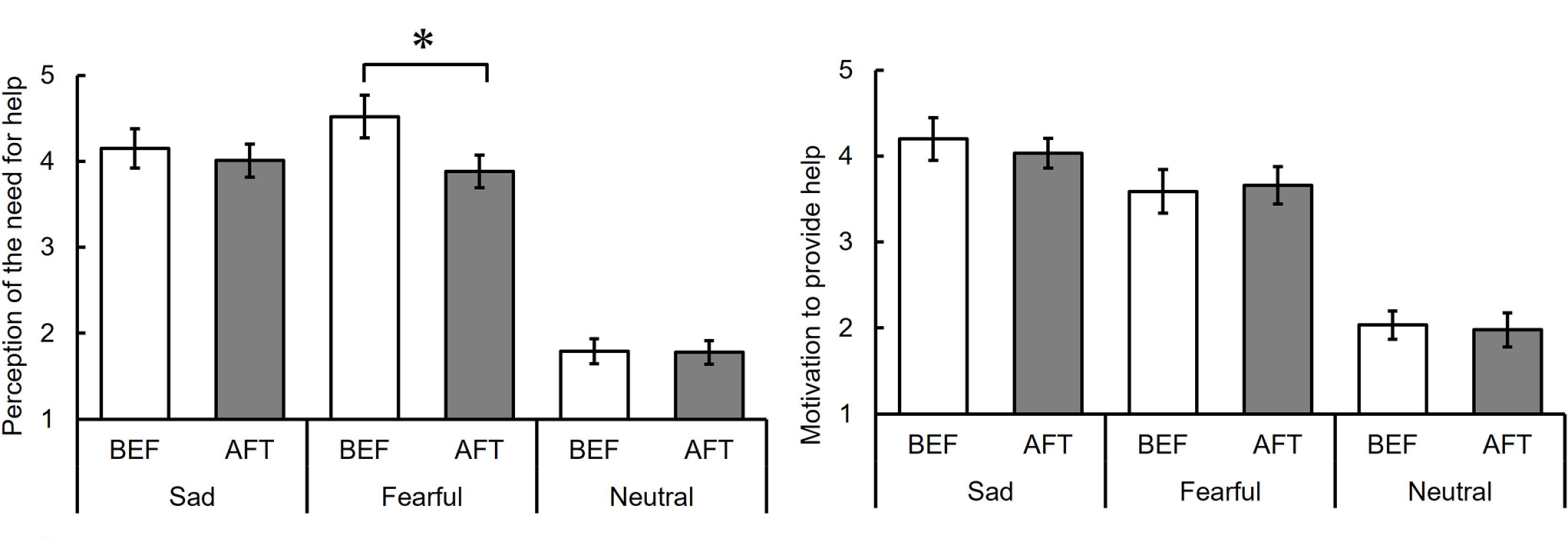
Ratings of the perception of the need for help and motivation to help before (BEF) and after (AFT) adaptation to fearful facial expressions for sad, fearful, and neutral facial expressions (Experiment 2). Note: The error bar indicates standard error. The asterisk indicates significant differences between BEF and AFT

Figure 5 displays the average ratings of the perceived need to help individuals exhibiting sad, fearful, and neutral facial expressions (see also Table S4). A two-way repeated measures ANOVA with phase (BEF vs. AFT) and facial expression (sad vs. fearful vs. neutral) revealed a significant main effect of facial expression, *F* (2, 34) = 100.69, *p <*.001, η_p_^2^ = 0.86, and a significant interaction, *F* (2, 34) = 7.34, *p* =.002, η_p_^2^ = 0.30. Follow-up analyses showed that the ratings of the need to help individuals with fearful facial expressions in the AFT phase significantly decreased from the BEF phase, *F* (1, 51) = 13.36, *p* <.001, whereas the ratings for individuals with sad facial expressions did not differ between the two phases *F*, (1, 51) = 0.63, *p* =.43. The main effect of the phase was not significant, *F* (1, 17) = 3.37, *p =*.08, η_p_^2^ = 0.17

### Motivation to provide help

Figure 5 also illustrates the average ratings of motivation to provide help individuals displaying sad, fearful, and neutral facial expressions (see also Table S4). A two-way repeated measures ANOVA with phase (BEF vs. AFT) and facial expression (sad vs. fearful vs. neutral) showed a significant main effect of facial expression: *F* (2, 34) = 52.56, *p <*.001, η_p_^2^ = 0.76. Subsequent multiple comparisons revealed that the ratings for neutral facial expressions were lower than those for sad and fearful facial expressions: *t* (34) = 9.80, *p <*.001, *r* =.86 and *t* (34) = 7.52, *p* <.001, *r* =.79, respectively; and the ratings for sad expressions were higher than those for fearful expressions: *t* (34) = 2.28, *p* =.03, *r* =.36. The main effect of the phase and the interaction between the phase and facial expression were not significant: *F* (1, 17) = 0.16, *p* =.70, η_p_^2^ = 0.01 and *F* (2, 34) = 0.61, *p =*.55, η_p_^2^ = 0.03, respectively. These results suggest that adaptation to fearful facial expressions did not influence the motivation to provide help.

## Discussion

We investigated the impact of adapting to fearful facial expressions on judgments of helping (i.e., perceived need for help and motivation to provide help) for sad and fearful faces. The ratings for the perceived need for help decreased only for fearful faces following adaptation, while the ratings for sad faces remained consistent pre- and post-adaptation. The results could be explained by the facial expression adaptation studies, which found that the aftereffect selectively suppressed recognition of facial expressions from the same category as the adapted faces (Hsu & Young, 2004; Juricevic & Webster, 2012). Thus, these findings suggest a decrease in the evaluation of the need for help due to a reduction in sensitivity to fearful facial expressions. Regarding the motivation to provide help, the ratings for sad faces were higher than those for fearful faces but remained unaffected by adaptation to fearful faces. This is in line with previous findings (Nakashima et al., 2009).

To directly compare the aftereffects of sad and fearful faces on helping judgment, we conducted a mixed-design ANOVA with experiment (between-participant factor; Experiments 1 and 2), phase (within-participant factor; BEF vs. AFT), and facial expression (within-participant factor; sad vs. fearful vs. neutral). For the ratings of the perceived need for help, the main effects of phase and facial expressions, as well as the interactions of phase × facial expression and experiment × phase × facial expression, were significant: *F* (1, 34) = 8.94, *p* =.005, η_p_^2^ = 0.21, *F* (2, 68) = 195.85, *p* <.001, η_p_^2^ = 0.85, *F* (2, 68) = 5.01, *p* =.009, η_p_^2^ = 0.13, and *F* (2, 68) = 3.51, *p* =.04, η_p_^2^ = 0.09, respectively. As detailed in the Results section of each experiment, the simple interaction of phase × facial expression was not significant in Experiment 1, but was significant in Experiment 2. This suggests that adaptation to sad faces influenced the perception of the need for help for both sad and fearful faces, whereas adaptation to fearful faces influenced this perception only for fearful faces.

It is possible that the intensity of the facial expressions used as adaptation stimuli in Experiments 1 and 2 influenced their aftereffects, as the aftereffect is reportedly stronger with more intense facial expressions (Minemoto et al., 2022; Skinner & Benton, 2010). We compared the intensity of sadness in the adaptation stimuli in Experiment 1 and the intensity of fear in the adaptation stimuli in Experiment 2 using an unpaired t-test. The results showed no significant difference between the adaptation stimuli in Experiments 1 and 2, *t* (34) = 0.13, *p* =.45, *r* =.02, indicating that the asymmetric results of Experiments 1 and 2 were not due to differences in the intensities of the adaptation stimuli.

Regarding the ratings of motivation to provide help, a mixed-design ANOVA was conducted with experiment, phase, and facial expression. The results showed that only the main effect of facial expression was significant, *F* (2, 68) = 79.63, *p* <.001, η_p_^2^ = 0.70. Multiple comparisons indicated that the ratings for sad facial expressions were the highest, followed by fear and neutral expressions, *t*s (68) ≥ 2.84, *p <*.001, *r*s *≥* 0.33, irrespective of the experiment and adaptation phases.

The asymmetric results of Experiments 1 and 2 may reflect the differing relationships between each facial expression and helping judgment. Adaptation to sad facial expressions not only suppressed sensitivity to sadness but also the perception mechanism of the need for help. Given that the adaptation aftereffect is automatic and unintended for the perceiver, rather than intentional, we infer that the perception mechanism of the need for help is triggered when we encounter sad facial expressions. This automatic induction, however, does not apply to fearful facial expressions although the need for help is related to both sad and fearful facial expressions.

### Experiment 3

Experiment 1 demonstrated a decrease in the ratings of perceived need for help following adaptation to sad facial expressions, irrespective of the test facial expressions. Considering that adaptation to fearful facial expressions reduced the ratings of perceived need for help for the same facial expression in Experiment 2, the results of Experiments 1 and 2 suggest that only adaptation to sad facial expressions suppress the perception of the need for help for both sad and fearful facial expressions. However, it is possible that those results were influenced by the characteristics of the rating task, which allows for a more detailed observation of changes in intensity (as we can observe changes in intensity within categories) compared to the category forced-choice task used in previous studies (Hsu & Young, 2004; Juricevic & Webster, 2012). A previous study noted that the rating task is more sensitive to changes in intensity than the forced-choice task (Burton et al., 2016). In other words, the possibility remains that adaptation to sad facial expressions does not alter the categorical perception of other negative expressions such as fear, but it could influence the perceived intensity, and this effect can be detectable through an intensity-rating task. To investigate this, we conducted Experiment 3, comparing the intensity of sadness before and after adaptation using the rating task. If the findings of Experiments 1 and 2 were due to a change in sensitivity to sadness (i.e., facial expression-dependent), adaptation to sad facial expressions would result in a decrease in the intensity of sadness for both sad and fearful facial expressions.

## Method

### Participants

Eighteen undergraduate and graduate students (including eight women; mean age 22.1; SD = 3.1) with normal or corrected-to-normal vision participated. All participants were naïve to the experiment’s purpose prior to participation. All participants provided signed consent prior to the experiment, which adhered to the principles of the Declaration of Helsinki.

### Apparatus and stimuli

The apparatus and stimuli were identical to those in Experiment 1.

### Procedure

The procedure mirrored that of Experiment 1, except that only “intensity of sadness” and “intensity of fear” were utilized as rating items during both the BEF and AFT phases. No other rating items were employed.

### Analysis

The analysis followed the same pattern as in Experiment 1, with two exceptions. First, the dependent variables were the average rating scores for the intensity of sadness and fear facial expressions during the BEF and AFT phases. Second, a two-way repeated measures ANOVA was conducted, considering the independent variables of phase (BEF vs. AFT) and facial expression (sad vs. fearful vs. neutral).

### Data collection and availability

The raw data for this experiment can be found at https://osf.io/3zrfb/. The materials are available upon request to the corresponding author, as they contain personal information (faces).

## Results

**Fig. 6:**
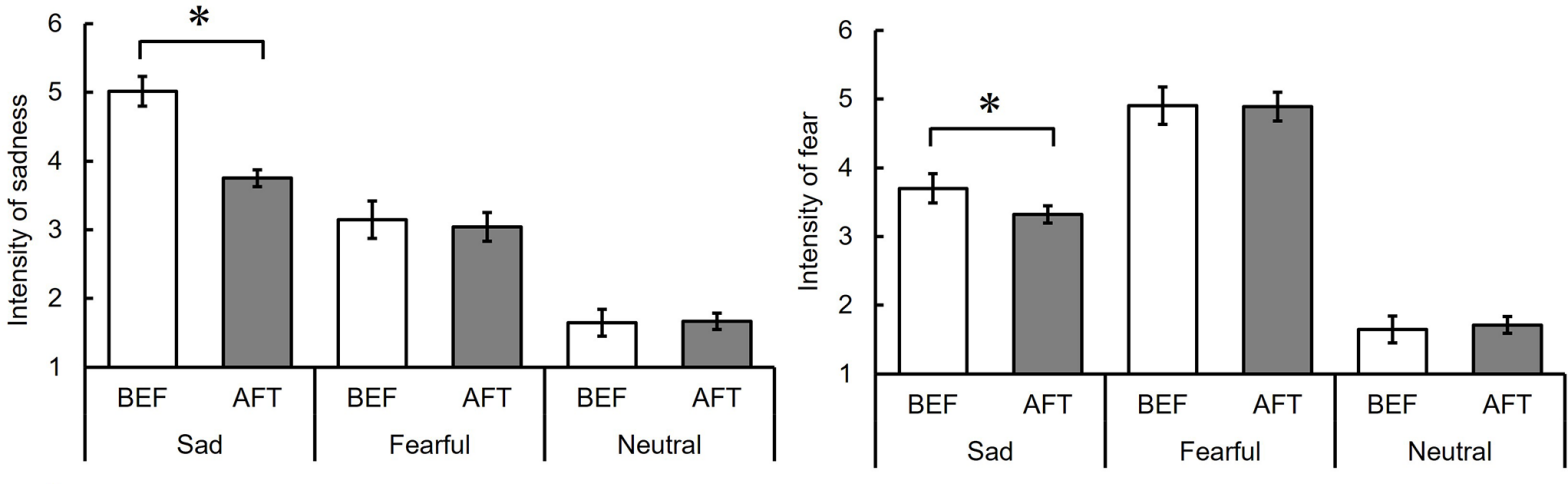
Intensity ratings of sadness and fear before (BEF) and after (AFT) adaptation to sad facial expressions for neutral, sad, and fearful facial expressions (Experiment 3). Note: The error bar indicates standard error. The asterisk indicates significant differences between BEF and AFT

Figure 6 presents the average ratings of the intensity of sadness and fear for each facial expression (neutral, sad, and fearful) (see also Table S5)

### Intensity of sadness

The two-way repeated measures ANOVA revealed significant main effects for both phase (BEF vs. AFT) and facial expression (sad vs. fearful vs. neutral), *F* (1, 17) = 8.96, *p =*.008, η_p_^2^ = 0.35, *F* (2, 34) = 66.01, *p* <.001, η_p_^2^ = 0.80, respectively, and a significant interaction between them, *F* (2, 34) = 12.30, *p* <.001, η_p_^2^ = 0.42. The ratings for sad facial expressions decreased after adaptation, *F* (1, 51) = 32.13, *p* <.001, while those for fearful and neutral facial expressions remained comparable before and after adaptation, *F* (1, 51) = 0.22, *p* =.64, *F* (1, 51) = 0.01, *p* =.92. Sad facial expressions received the highest ratings, followed by fearful and neutral expressions, in both the BEF, *t*s (68) ≥ 5.41, *p* <.001, *r*s ≥ 0.55, and AFT phases, *t*s (68) ≥ 2.57, *p* <.05, *r*s ≥ 0.30.

### Intensity of fear

Another two-way repeated measures ANOVA showed a significant main effect of facial expression and a significant interaction, *F* (2, 34) = 144.18, *p* <.001, η_p_^2^ = 0.89 and *F* (2, 34) = 3.61, *p =*.04, η_p_^2^ = 0.18, respectively. However, the main effect of phase was not significant, *F* (1, 17) = 1.59, *p =*.22, η_p_^2^ = 0.09. The ratings for sad facial expressions decreased after adaptation, *F* (1, 51) = 8.02, *p* =.007, while those for fearful and neutral facial expressions remained comparable before and after adaptation, *F* (1, 51) = 0.02, *p* =.90 and *F* (1, 51) = 0.25, *p* =.62, respectively. Fearful facial expressions received the highest ratings, followed by sad and neutral expressions, in both the BEF, *t*s (68) ≥ 5.75, *p* <.001, *r*s ≥ 0.57, and AFT phases, *t*s (68) ≥ 7.48, *p* <.001, *r*s ≥ 0.67.

## Discussion

The study investigated whether adaptation to sad facial expressions reduces the perceived intensity of sadness and fear. Although intensity ratings of sadness for sad facial expressions decreased after adaptation to sad facial expressions, ratings for fearful and neutral facial expressions remained comparable before and after adaptation. Importantly, intensity ratings of fear also showed the same pattern, in which ratings of fear for sad facial expressions only decreased. Taken together, the results indicated that adaptation to sad facial expressions suppressed the recognition of sad facial expression solely, but not other negative expressions. This finding is inconsistent with the results of Experiment 1, which showed that aftereffect of sad facial expressions affected the ratings of perception of the need for help on both sad and fearful facial expressions. These results indicated that the results of Experiment 1 were not due to a change in sensitivity to sad facial expressions, and that participants adapted to the social signals themselves.

### Experiment 4

Observing sad facial expressions can induce corresponding moods and feelings (Schneider et al., 1994; Wild et al., 2001), potentially altering cognitions; notably, a sad mood can enhance the accuracy of facial expression recognition (Chepenik et al., 2007; however, sad mood induction facilitates the accuracy of facial expression recognition in their study). Therefore, moods induced by adaptation stimuli, such as sad facial expressions, may influence helping judgments. To investigate this, we replicated the rating task from Experiment 1, replacing the facial adaptation paradigm with a sad mood induction.

## Method

### Participants

Eighteen undergraduate and graduate students (including eight women; mean age 21.6; SD = 3.4) with normal or corrected-to-normal vision participated in the experiment. They were naïve to the experiment’s purpose and signed consent forms beforehand. They were informed during recruitment and consent that they would need to recall personal sad events during the experiment. The Ethics Committee at the Unit for Advanced Studies of the Human Mind, Kyoto University (29-P-23), approved this study.

### Apparatus and stimuli

The apparatus and stimuli were identical to those in Experiment 1, except for the use of SuperLab 5.0.5 software (Cedrus) to design and control the experiment.

### Procedure

In this experiment, the ADP phase was replaced with a Mood Induction (MI) phase, and a recovery phase was added after the AFT phase. The CON and BEF phases were identical to those in Experiment 1. During the MI phase, participants were asked to recall and describe their two saddest life events as vividly as possible on an A4-sized paper from a first-person perspective, as if writing a diary entry, for 10 min. The time allocation for describing the two events was left to the participants. They were then instructed to vividly and firmly visualize the saddest event (or the second saddest if the first was too difficult to recall) for a minute (Culot et al., 2021). The experimenter was not present in the room during this process.

In the AFT phase, a black cross was displayed for five seconds instead of an adaptation face. Participants were asked to vividly recall the sad event visualized in the MI phase while viewing the cross. To alleviate participants’ mood in the recovery phase, a soothing animal movie accompanied by relaxing music was shown for approximately 2 min (Culot et al., 2021). To assess participants’ emotions, we used the Self-Assessment Manikin (SAM, © Peter J. Lang 1994), which consists of five illustrations ranging from happy to unhappy (Bradley & Lang, 1994; Lang, 1980). Participants were asked to mark the most appropriate point on a 9-point scale beneath or between each illustration. This was conducted four times: after the BEF, MI, AFT, and recovery phases. Additionally, after visualizing the sad image in the MI phase, participants rated their success in creating the image on a 9-point scale, with questions such as “Did you create the image well?”, “Did you create the image vividly?”, and “Did you recreate the image as you experienced it?”, with a 9-point scale ranging from 1 (not at all) to 9 (very well) to check whether they could make the images as instructed.

### Analysis

The analysis of intensity of sadness and fear, perceived need for help, and motivation to provide help followed the same procedures as in Experiment 1. For the manipulation check, we used the SAM ratings from each phase, with 1 representing unhappiness and 9 representing happiness. These ratings served as dependent variables. We conducted a one-way repeated measures ANOVA with a dependent variable of phase for four phase levels (after BEF, MI, AFT, and recovery) to verify the induction of a sad mood in participants. We calculated the *Mean* ± 3*SD* for the standard error of the mean in each phase and each item in the questionnaire regarding the creation of the sad image. This was done to exclude participants who had difficulties and outliers with the experimental manipulation.

### Data collection and availability

The raw data for this experiment can be found at https://osf.io/3zrfb/. Materials are available upon request to the corresponding author, as they contain personal information (faces).

## Results

### Manipulation check

**Fig. 7:**
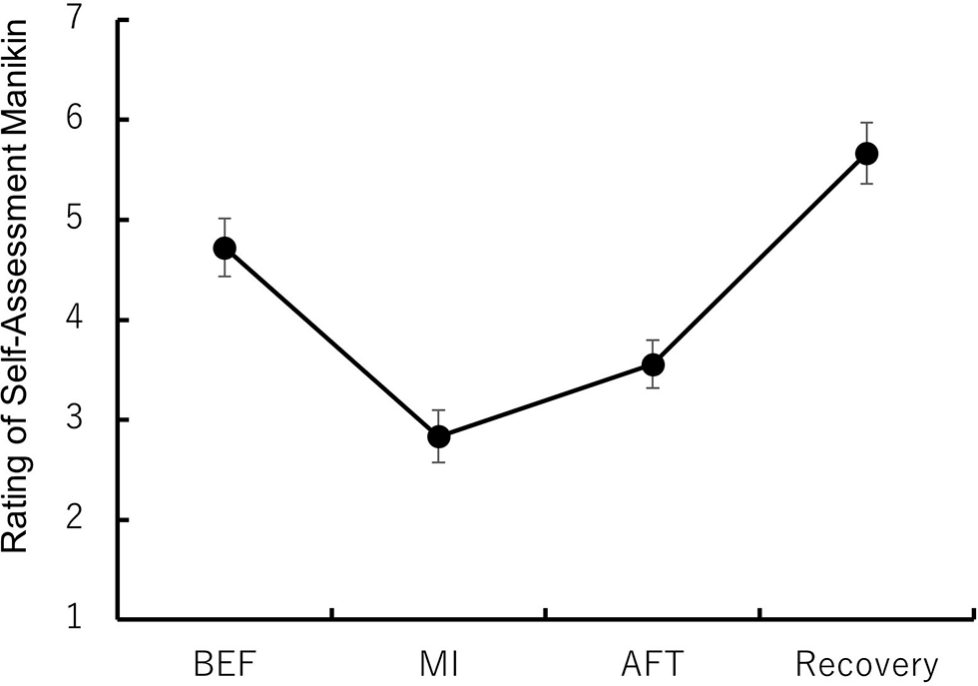
Ratings of the Self-Assessment Manikin (SAM) before mood induction (BEF), mood induction (MI), after mood induction (AFT), and recovery. Note: The error bar indicates the standard error

For each phase and item in the SAM questionnaire related to the sad image, all participants’ ratings fell within the range of *Mean* ± 3*SD*. Figure 7 presents the average ratings of SAM in each phase (see also Table S6). A one-way repeated measures ANOVA revealed a significant main effect of phase, *F* (3, 51) = 28.24, *p* <.001, η_p_^2^ = 0.71. Multiple comparisons using Ryan’s method showed significant differences between each phase. The recovery phase had the highest ratings, followed by BEF, AFT, and MI, *t*s (51) ≥ 2.62, *p* <.05, *r*s *≥*. 34. Participants rated their ability to create the image well at an average of 6.61 (SD = 1.77), the vividness of their image at 6.44 (SD = 1.67), and their experience of imagining it at 5.17 (SD = 1.83). These results suggest that participants successfully created a sad image, inducing a sad mood.

### Intensity of sadness and fear

**Fig. 8:**
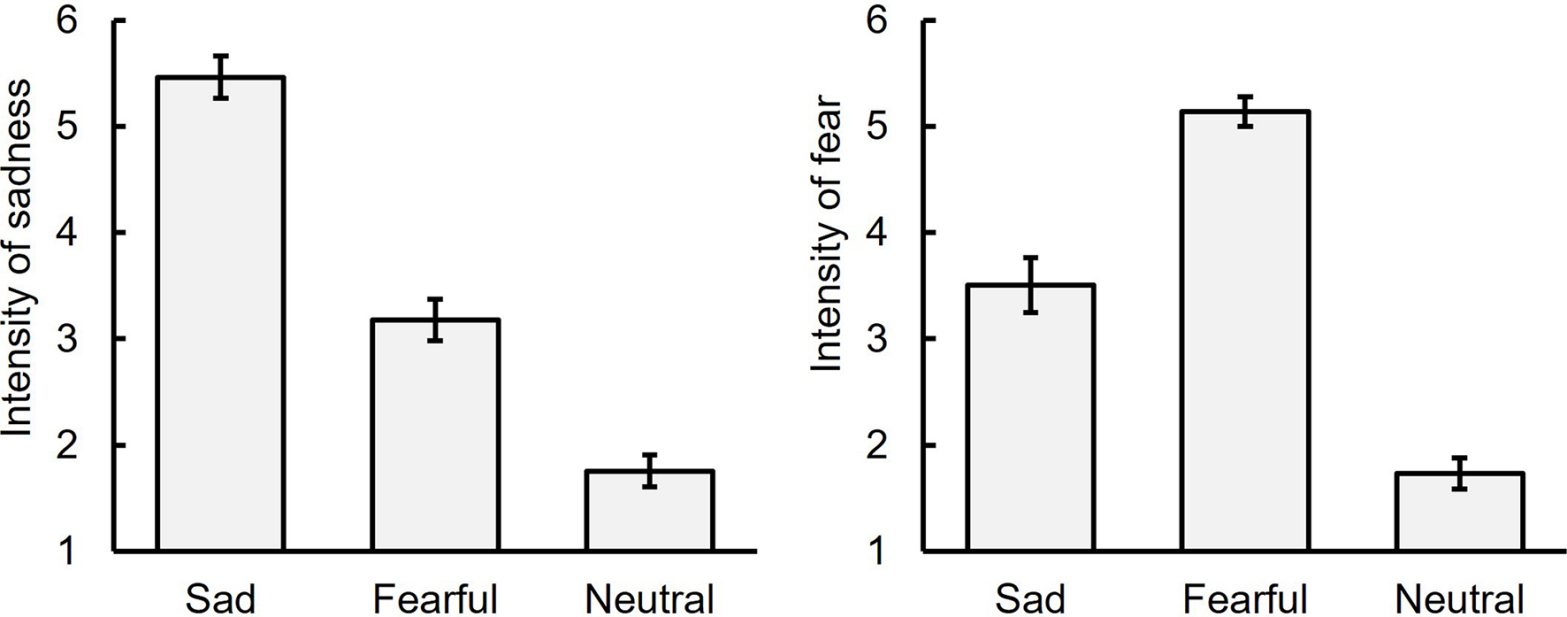
Intensity ratings of sadness and fear for sad, fearful, and neutral facial expressions before the sad mood induction phase (Experiment 4). Note: The error bar indicates the standard error

Figure 8 displays the average ratings of sadness and fear intensity for each facial expression (neutral, sad, and fearful) in the BEF phase (see also Table S7). For sadness intensity, a one-way repeated measures ANOVA showed a significant main effect of facial expression, *F* (2, 34) = 128.87, *p* <.001, η_p_^2^ = 0.88. Multiple comparisons using Ryan’s method revealed that the rating for sad facial expressions was the highest, followed by fearful and neutral expressions, *t*s (34) ≥ 6.11, *p* <.001, *r*s *≥*. 72.

For fear intensity, a one-way repeated measures ANOVA showed a significant main effect of facial expression, *F* (2, 34) = 109.63, *p* <.001, η_p_^2^ = 0.87. Multiple comparisons revealed that the rating for fearful facial expressions was the highest, followed by sad and neutral expressions, *t*s (34) ≥ 7.10, *p* <.001, *r*s ≥ 0.77. These results suggest that participants accurately recognized the models’ facial expressions.

### Perception of the need for help

**Fig. 9:**
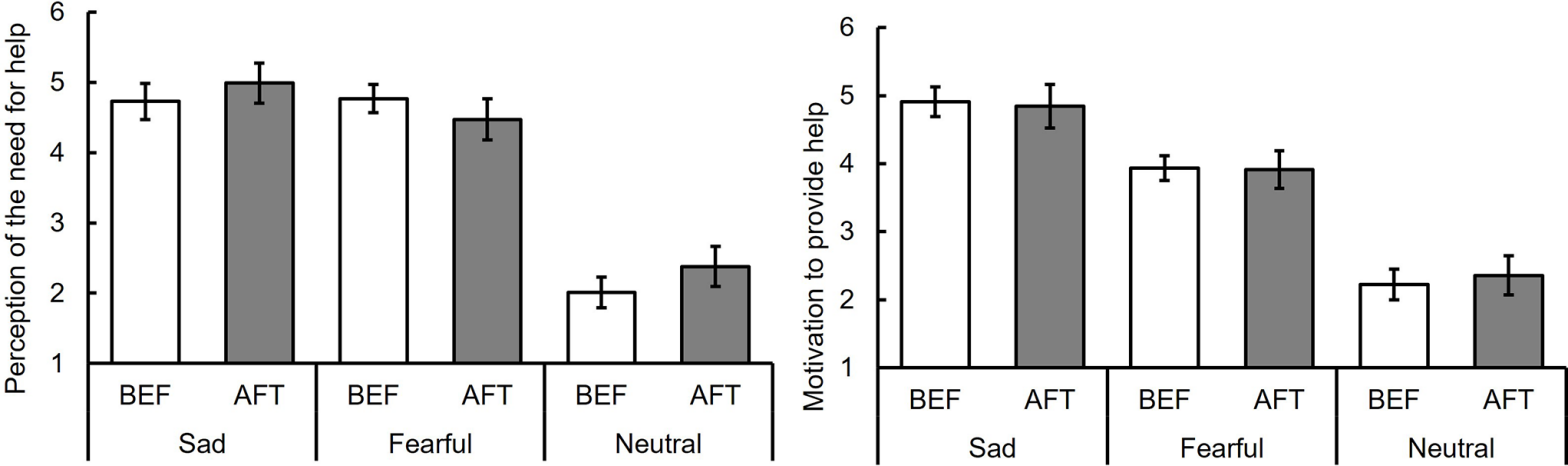
The ratings of the perception of the need for help and motivation to help before (BEF) and after (AFT) sad mood induction for sad, fearful, and neutral facial expressions (Experiment 4). Note: The error bar indicates the standard error

Figure 9 presents the average ratings of the perceived need for help for individuals with sad, fearful, and neutral facial expressions (see also Table S8). A two-way repeated measures ANOVA with phase (BEF vs. AFT) and facial expression (sad vs. fearful vs. neutral) revealed a significant main effect of facial expression, *F* (2, 34) = 71.63, *p* <.001, η_p_^2^ = 0.81. Subsequent multiple comparisons showed that the ratings for sad and fearful facial expressions were higher than those for neutral expressions, *t* (34) = 10.82, *p* <.001, *r* =.88 and *t* (34) = 9.85, *p* <.001, *r* =.86, respectively. The ratings for sad and fearful facial expressions were comparable, *t* (34) = 0.97, *p* =.34, *r* =.16. The main effects of phase and interaction between facial expression and phase were not significant; *F* (1, 17) = 0.38, *p* =.57, η_p_^2^ = 0.02 and *F* (2, 34) = 2.93, *p =*.07, η_p_^2^ = 0.15, respectively. These results suggest that the induction of a sad mood did not influence the perception of the need for help for individuals with both sad and fearful facial expressions.

### Motivation to provide help

Figure 9 also shows the average ratings of participants’ motivation to help individuals with sad, fearful, and neutral facial expressions. A two-way repeated measures ANOVA with phase (BEF vs. AFT) and facial expression (sad vs. fearful vs. neutral) revealed a significant main effect of facial expression, *F* (2, 34) = 54.21, *p* <.001, η_p_^2^ = 0.76. Subsequent multiple comparisons showed that the ratings for neutral facial expressions were lower than those for sad and fearful facial expressions, *t* (34) = 10.30, *p* <.001, *r* =.87 and *t* (34) = 6.50, *p* <.001, *r* =.74, respectively, and the ratings for sad expressions were higher than those for fearful expressions, *t* (34) = 3.80, *p* <.001, *r* =.55. The main effect of the phase and the interaction between the phase and facial expression were not significant, *F* (1, 17) < 0.01, *p* =.95, η_p_^2^ < 0.001 and *F* (2, 34) = 0.31, *p =*.73, η_p_^2^ = 0.02, respectively. The results align with Experiments 1 and 2 and suggest that the induction of a sad mood did not affect the motivation to provide help.

## Discussion

Experiment 4 was conducted to examine whether a sad mood influences helping judgments. The results showed comparable ratings before and after the induction of a sad mood. A post-hoc mixed-design ANOVA was conducted to directly compare the effects of adaptation and induction to sad facial expressions on helping judgment. This analysis involved experiment (a between-participant factor; Experiment 1 vs. Experiment 4), phase (a within-participant factor; BEF vs. AFT), and facial expression (a within-participant factor; sad vs. fearful vs. neutral).

For the ratings of perceived need for help, the main effects of the experiment and facial expression were significant, *F* (1, 34) = 4.67, *p* =.04, η_p_^2^ = 0.12, and *F* (2, 68) = 163.48, *p* <.001, η_p_^2^ = 0.83, respectively. The interaction between experiment and phase was marginally significant, *F* (1, 34) = 3.07, *p* =.06, η_p_^2^ = 0.10. Further analysis revealed that the perception of the need for help decreased after adaptation in Experiment 1, regardless of facial expression, *F* (1, 34) = 4.35, *p* =.04. However, this was not the case after MI in Experiment 4, *F* (1, 34) = 0.40, *p* =.53. The main effects of phase and other interactions were not significant.

Given that the number of participants in Experiment 4 was the same as in Experiment 1, there may have been insufficient power to conduct an analysis comparing adaptation and expression induction. However, our results showed a trend consistent with our hypothesis, suggesting that the results of Experiment 1 cannot be fully explained by MI due to the adaptation of sad facial expressions. For the ratings of motivation to provide help, a mixed-design ANOVA with experiment, phase, and facial expression revealed that only the main effect of facial expression was significant, *F* (2, 68) = 83.40, *p* <.001, η_p_^2^ = 0.71. Multiple comparisons revealed that the ratings for sad facial expressions were the highest, followed by fear and neutral expressions, *t*s (68) ≥ 3.88, *p* <.001, *r*s *≥* 0.43, regardless of the experiments and phases.

## General discussion

This study investigated the representation of facial expressions and the social signals they evoke. In Experiments 1 and 2, we compared the perception of the need for help and the motivation to help others displaying sad and fearful facial expressions before and after adaptation to these expressions. Experiment 1 revealed that after viewing sad facial expressions, ratings of the need for help decreased for both sad and fearful expressions. In contrast, Experiment 2 demonstrated that ratings decreased only for fearful expressions after viewing fearful expressions. Experiments 3 and 4 aimed to explore other possible explanations for the asymmetric results of Experiments 1 and 2. Experiment 3 examined whether the sensitivity to fearful expressions was suppressed by adaptation to sad expressions in the rating task. The results showed that only the sensitivity to the recognized intensities of sad expressions was reduced, not to fearful expressions. This indicates that the common decrease in the perception of the need for help for both expressions after adaptation to sad expressions was not due to a decrease in sensitivity to fearful expressions. Experiment 4 investigated whether the mood induced by viewing sad adaptation faces affected the perception of the need for help. The results showed no change in the ratings of the need for help for sad and fearful expressions before and after inducing a sad mood. Furthermore, the results of Experiment 4 indicated that the decreased ratings in Experiments 1, 2, and 3 after adaptation were not due to the practice effect of presenting the same stimuli twice, as the ratings of Experiment 4 were comparable between the first and second presentations.

The results suggest that while sad and fearful facial expressions are factors involved in judging the need to help another person, which can be observed in direct rating task of altruism or helping (Marsh & Ambady, 2007; Nakashima et al., 2009), there is a difference in social function between these expressions. The finding that adaptation to fearful expressions affected the perception of the need for help only for fearful expressions suggests that adapting to fearful expressions suppresses sensitivity to the same expression, leading to a decrease in the ratings of the need to help people with fearful expressions. This is consistent with previous adaptation studies showing that the aftereffect of facial expression was observed when the adaptation and test stimuli belonged to the same emotion category (Hsu & Young, 2004; Juricevic & Webster, 2012; Webster et al., 2004)^3^. In contrast, the result that adaptation to sad expressions affected the perception of the need to help for both sad and fearful expressions is inconsistent with the previous adaptation studies. These contradictory results with previous study suggest that adaptation to sad expressions not only suppresses sensitivity to sad expressions but also the perception mechanism of the need for help. In addition, this suppression was induced by the passive viewing. Taken together, it suggests that sad facial expressions automatically trigger the perception of the need for help when one sees them, but it is not the case with fearful facial expressions, despite the fact that both expressions convey the impression of need for help. In conclusion, the need for help can be represented as a facial expression-independent manner only when sad expressions were presented, but this does not apply to fearful expressions.

Regarding motivation to provide help, we found no significant impact of adaptation to both sad and fearful facial expressions, nor the induction of a sad mood. This suggests that factors other than sensitivity to facial expressions are responsible for activating the motivation to help. For example, Takagi (1997) proposed a model that incorporates the benefits and costs of providing help in the decision-making process when individuals notice another person in distress. Notably, this result does not negate the contribution of facial expressions to the motivation to help. Indeed, the motivation to help was highest for individuals with sad facial expressions, followed by those with fearful and neutral expressions (albeit marginally in Experiment 1). This might be influenced more by the category of facial expressions rather than their intensity. Our findings suggest that the recognition of facial expressions, the perception of social signals from faces, and the motivation to help others do not entirely share the same underlying mechanisms.

This study demonstrated that the adaptation method is useful for inferring the neural mechanisms of social signals derived from facial expressions and for exploring the types of social functions automatically induced by each facial expression. Previous studies have employed an adaptation paradigm to reveal the recognition system of visual stimuli (Clifford & Rhodes, 2005; Webster, 2011). This paradigm offers the advantage of systematic examination as it allows for the control of specific visual information by eliminating other influences. Adaptation is caused by the exhaustion of the responses of nerve cells that process adaptive stimuli. As observed in this study, the fact that adaptation to facial expressions alters social cognition independently of the sensitivity to facial expression recognition suggests that facial expressions are closely and unintentionally linked to the neuronal groups involved in the cognitive processing of helping behaviors.

Accurate recognition of situations is crucial in social life. This study indicated that facial expressions have a social function that is automatically triggered when these expressions are seen. If this function underlies daily communication, there is a potential issue where, although facial expressions can be correctly recognized, the automatic social signals may not be perceived, leading to communication difficulties. Therefore, it is important to explore the social signals associated with each facial expression to deepen our understanding of the social role of facial expressions. For example, considering that fear expressions are displayed in highly urgent or life-threatening situations, they may preferentially carry social signals that are appropriate for such contexts. The limitation of this study is that we assessed helping judgment using a Likert scale and a gap may exist between ratings and actual behavior. In addition, as each experiment involved different participants, our results did not indicate a direct link between perceived intensity of facial expressions and the change in perception of the need for help. Finally, though our results have suggested the facial expression-independent social signal, they were insufficient to discuss why the same social signals were expressed differently in sad and fearful facial expressions. Future research is needed to reveal the social functions of facial expressions in actual behavioral situations (i.e., situations where individuals have to face potential costs for providing help).

In summary, our study suggested the independent representation of social signals from that of facial expressions. Furthermore, the results indicated that, although both sad and fearful facial expressions are related to the perception of the need for help and the motivation to provide help, sad facial expressions automatically induce the need for help, but fearful facial expressions do not have this automatic function. However, the motivation to help remains constant, regardless of the change in sensitivity to facial expressions. The adaptation paradigm will be a useful tool to reveal further social functions of facial expressions.

## Electronic supplementary material

Below is the link to the electronic supplementary material.

Supplementary Material 1

## Data Availability

The raw data for this experiment can be found at https://osf.io/3zrfb/. Materials are available upon request to the corresponding author, as they contain personal information (faces).

## Footnotes

1 The range of the rating values were as following: “perception of the need for help” from 1 (I never think so) to 7 (I absolutely think so), “motivation to provide help” from 1 (I never want to do) to 7 (I absolutely want to do), “intensity of sadness” from 1 (She never expresses sadness) to 7 (She absolutely expresses sadness), and “intensity of fear” from 1 (She never expresses fear) to 7 (She absolutely expresses fear). The other two items were concerning to the subjective positive and negative feelings.

2 To confirm whether the number of participants was sufficient, we conducted a post-hoc power analysis, and the result showed 1-β = 0.79.

3 These studies used category selection tasks (Hsu & Young, 2004; Juricevic & Webster, 2012) and 2AFC (Webster et al., 2004) instead of rating task which this study used, but all of them showed that the same expression as the adapted expression was less likely to be selected.
