# Supplementary material for: Facial expression adaptation impairs perceived social signal across expressions
Source: Psychol Res. 2025 Mar 24;89(2):73. doi: 10.1007/s00426-025-02094-4 (PMC11933154; doi:10.1007/s00426-025-02094-4)
Supplement: Supplementary file 1 — Supplementary Material 1 [file 426_2025_2094_MOESM1_ESM.pdf]

Supplementary Materials

Table S1. *Intensity Ratings of Sadness and Fear for Each Facial Expression Before the Adaptation (Experiment 1)*

	Intensity of Sadness			Intensity of Fear		
	Sad	Fearful	Neutral	Sad	Fearful	Neutral
Mean	5.08	3.04	1.51	3.34	5.07	1.53
S.E.	0.21	0.22	0.16	0.29	0.21	0.18

Note. *S.E.* indicates *standard error*.

Table S2. *Ratings of the Perception of the Need for Help and Motivation to Provide Help (Experiment 1)*

	Perception of the Need for Help						Motivation to Provide Help					
	Sad		Fearful		Neutral		Sad		Fearful		Neutral	
	BEF	AFT	BEF	AFT	BEF	AFT	BEF	AFT	BEF	AFT	BEF	AFT
Mean	4.36	3.80	4.69	4.32	1.76	1.59	4.42	4.19	3.73	3.79	1.88	2.13
S.E.	0.91	0.53	1.15	0.89	0.83	0.52	1.19	0.68	1.20	1.08	1.01	1.19

Note. *S.E.* indicates *standard error*. BEF and AFT indicate before and after the adaptation, respectively.

Table S3. *Intensity Ratings of Sadness and Fear for Each Facial Expression Before the Adaptation (Experiment 2)*

	Intensity of Sadness			Intensity of Fear		
	Sad	Fearful	Neutral	Sad	Fearful	Neutral
Mean	4.61	3.07	1.52	3.64	4.91	1.56
S.E.	0.16	0.20	0.14	0.21	0.23	0.15

Note. *S.E.* indicates *standard error*.

Table S4. *Ratings of the Perception of the Need for Help and Motivation to Provide Help (Experiment 2)*

	Perception of the Need for Help						Motivation to Provide Help					
	Sad		Fearful		Neutral		Sad		Fearful		Neutral	
	BEF	AFT	BEF	AFT	BEF	AFT	BEF	AFT	BEF	AFT	BEF	AFT
Mean	4.15	4.01	4.52	3.88	1.79	1.78	4.20	4.03	3.59	3.66	2.03	1.98
S.E.	0.98	0.81	1.06	0.80	0.62	0.59	1.05	0.73	1.08	0.92	0.69	0.83

Note. *S.E.* indicates *standard error*. BEF and AFT indicate before and after the adaptation, respectively.

Table S5. *Intensity Ratings of Sadness and Fear for Each Facial Expression Before and After the Adaptation (Experiment 3)*

	Intensity of Sadness						Intensity of Fear					
	Sad		Fearful		Neutral		Sad		Fearful		Neutral	
	BEF	AFT	BEF	AFT	BEF	AFT	BEF	AFT	BEF	AFT	BEF	AFT
Mean	5.02	3.75	3.14	3.04	1.64	1.67	3.70	3.32	4.91	4.89	1.64	1.71
S.E.	0.70	0.80	1.42	1.24	0.63	0.59	0.88	0.84	0.97	1.11	0.56	0.54

Note. *S.E.* indicates *standard error*. BEF and AFT indicate before and after the adaptation, respectively.

Table S6. *Ratings of the Self-Assessment Manikin for Each Session (Experiment 3)*

	Rating of Self-Assessment Manikin			
	BEF	MI	AFT	Recovery
Mean	4.72	2.83	3.56	5.67
S.E.	0.29	0.26	0.24	0.30

Note. *S.E.* indicates *standard error*. BEF, MI and AFT indicate before, during, and after the mood induction, respectively.

Table S7. *Intensity Ratings of Sadness and Fear for Each Facial Expression Before the Mood Induction (Experiment 4)*

	Intensity of Sadness			Intensity of Fear		
	Sad	Fearful	Neutral	Sad	Fearful	Neutral
Mean	5.46	3.18	1.76	3.51	5.14	1.73
S.E.	0.20	0.20	0.15	0.26	0.14	0.15

Note. *S.E.* indicates *standard error*.

Table S8. *Ratings of the Perception of the Need for Help and Motivation to Provide Help (Experiment 4)*

	Perception of the Need for Help						Motivation to Provide Help					
	Sad		Fearful		Neutral		Sad		Fearful		Neutral	
	BEF	AFT	BEF	AFT	BEF	AFT	BEF	AFT	BEF	AFT	BEF	AFT
Mean	4.73	4.99	4.77	4.47	2.01	2.38	4.91	4.84	3.93	3.91	2.22	2.36
S.E.	0.25	0.29	0.20	0.29	0.22	0.29	0.22	0.32	0.18	0.28	0.23	0.29

Note. *S.E.* indicates *standard error*. BEF and AFT indicate before and after the adaptation, respectively.
